# Wearables for Measuring Health Effects of Climate Change–Induced Weather Extremes: Scoping Review

**DOI:** 10.2196/39532

**Published:** 2022-09-09

**Authors:** Mara Koch, Ina Matzke, Sophie Huhn, Hanns-Christian Gunga, Martina Anna Maggioni, Stephen Munga, David Obor, Ali Sié, Valentin Boudo, Aditi Bunker, Peter Dambach, Till Bärnighausen, Sandra Barteit

**Affiliations:** 1 Heidelberg Institute of Global Health Faculty of Medicine and University Hospital Heidelberg University Heidelberg Germany; 2 Charité - Universitätsmedizin Berlin, Institute of Physiology, Center for Space Medicine and Extreme Environment Berlin Berlin Germany; 3 Department of Biomedical Sciences for Health Università degli Studi di Milano Milan Italy; 4 Kenya Medical Research Institute Kisumu Kenya; 5 Centre de Recherche en Santé Nouna Burkina Faso; 6 Center for Climate, Health, and the Global Environment Harvard T.H. Chan School of Public Health Boston, MA United States; 7 Department of Global Health and Population Harvard T.H. Chan School of Public Health Boston, MA United States; 8 Africa Health Research Institute KwaZulu-Natal South Africa

**Keywords:** wearable, consumer-grade wearables, fitness trackers, climate change, heat, global health, public health, review, mobile phone

## Abstract

**Background:**

Although climate change is one of the biggest global health threats, individual-level and short-term data on direct exposure and health impacts are still scarce. Wearable electronic devices (wearables) present a potential solution to this research gap. Wearables have become widely accepted in various areas of health research for ecological momentary assessment, and some studies have used wearables in the field of climate change and health. However, these studies vary in study design, demographics, and outcome variables, and existing research has not been mapped.

**Objective:**

In this review, we aimed to map existing research on wearables used to detect direct health impacts and individual exposure during climate change–induced weather extremes, such as heat waves or wildfires.

**Methods:**

We conducted a scoping review according to the PRISMA-ScR (Preferred Reporting Items for Systematic Reviews and Meta-Analyses extension for Scoping Reviews) framework and systematically searched 6 databases (PubMed [MEDLINE], IEEE Xplore, CINAHL [EBSCOhost], WoS, Scopus, Ovid [MEDLINE], and Google Scholar). The search yielded 1871 results. Abstracts and full texts were screened by 2 reviewers (MK and IM) independently using the inclusion and exclusion criteria. The inclusion criteria comprised studies published since 2010 that used off-the-shelf wearables that were neither invasive nor obtrusive to the user in the setting of climate change–related weather extremes. Data were charted using a structured form, and the study outcomes were narratively synthesized.

**Results:**

The review included 55,284 study participants using wearables in 53 studies. Most studies were conducted in upper–middle-income and high-income countries (50/53, 94%) in urban environments (25/53, 47%) or in a climatic chamber (19/53, 36%) and assessed the health effects of heat exposure (52/53, 98%). The majority reported adverse health effects of heat exposure on sleep, physical activity, and heart rate. The remaining studies assessed occupational heat stress or compared individual- and area-level heat exposure. In total, 26% (14/53) of studies determined that all examined wearables were valid and reliable for measuring health parameters during heat exposure when compared with standard methods.

**Conclusions:**

Wearables have been used successfully in large-scale research to measure the health implications of climate change–related weather extremes. More research is needed in low-income countries and vulnerable populations with pre-existing conditions. In addition, further research could focus on the health impacts of other climate change–related conditions and the effectiveness of adaptation measures at the individual level to such weather extremes.

## Introduction

### Background

Climate change is one of the biggest global health threats of the century [1], and the field of climate and health research has been rapidly growing [2]. Many environmental conditions such as rising temperatures, floods, wildfires, heat waves, droughts, and other extreme weather events can be linked to climate change according to the 2021 Intergovernmental Panel on Climate Change report [3] and may, directly and indirectly, impact human health [4]. The wide-ranging health effects of these weather extremes include malnutrition from food insecurity; infectious disease; respiratory, cardiovascular, neurological, and mental health disorders; and mortality [4,5].

Epidemiological studies often focus on the relationship between heat and mortality or morbidity in terms of the number of hospital admissions or long-term effects but do not consider individual exposure and direct health effects [5]. Furthermore, most studies use weather and climate data from satellites or the nearest weather station, which is often located at the airport. These approaches do not consider granular spatial and temporal differences in weather exposure or individual factors that influence the exposure such as time spent indoors [6,7]. To this end, consumer-grade wearable devices (hereafter *wearables*) could generate high-resolution data at the individual level, measuring exposure and health parameters in the real-life environment, the ecological momentary assessment [8]. Wearables can cover a variety of variables and physiological data, including, among others, activity levels, sleep, sweat rate, and heart rate (HR) [9], presenting a potential solution to the shortage of short-term and individual-level data in climate change and health research.

In recent years, some reviews have been conducted on the assessment of heat strain and individual heat exposure using wearable devices. However, these studies have mainly focused on urban and occupational heat exposure [10,11], although populations living in low- and middle-income countries and rural settings have a high vulnerability to climate change [12]. Although the urban heat island effect describes higher heat exposure in cities owing to human activities and dense concentrations of surfaces that absorb and retain heat, rural populations are often more exposed because of their reliance on climate-sensitive livelihoods [10,12]. Some reviews have examined the validity of various wearables but only in moderate climate settings [13,14]. Furthermore, many studies [15,16] used prototypes and not off-the-shelf devices, which make them difficult to reproduce in the field.

### Research Objectives

Therefore, the overarching objectives of this review were (1) to map the available research on the use of off-the-shelf wearables for measuring direct health effects of and individual exposure to climate change–induced weather extremes such as heat, (2) to examine current approaches to wearable use in this field, and (3) to identify gaps in the research. We particularly focused on (1) demographic characteristics, (2) selected wearable devices and their measures, (3) extreme weather condition exposure and data collection methods, (4) analytical approaches, (5) validity of wearables in extreme weather conditions, and (6) observed effects of extreme weather exposure on health (especially of heat on sleep, physical activity, and HR, as well as occupational heat stress).

## Methods

### Overview

The methodology for this scoping review was based on the framework outlined by Arksey and O'Malley [17] and Peters et al [18] and in accordance with the PRISMA-ScR (Preferred Reporting Items for Systematic Reviews and Meta-Analyses extension for Scoping Reviews) [19] (Multimedia Appendix 1). A review protocol can be obtained from the principal author (MK) upon request. A scoping review seemed most appropriate to approach the research objective, as initial research into this topic revealed a broad scope of heterogeneous studies, however, limited in their numbers.

### Eligibility Criteria

We included articles that were available in English and published after January 1, 2010, because wearables have become widely available on the consumer market and were also increasingly adopted in research since then [20,21]. Types of studies included were case studies, observational studies, non–randomized controlled trials, and randomized controlled trials. We included any consumer- or research-grade wearables that were available off-the-shelf, could be worn on the body, and were neither invasive nor obtrusive (excluding, eg, ingestible, handheld, or wired devices). All types of sensors or measurements that measured at least one physiological parameter or individual exposure were included. For a complete list of eligibility criteria, see Textbox 1.

**Inclusion and exclusion criteria.**

**Inclusion criteria**

- Publications:
  Full text available
  Published in the English language
  Published between January 2010 and September 2021
  Randomized controlled trials (RCTs), non-RCTs, observational studies, or case studies
- Wearable device:
  Off-the-shelf wearable electronic devices
  Noninvasive and nonobtrusive
  Measuring at least one physiological parameter (eg, heart rate or sleep duration) or individual exposure (eg, ambient temperature)
- Climate change:
  Climate change–related weather extremes: heat, flood, drought, wildfire, tropical cyclone, or heavy precipitation
  Exposure: outdoors, indoors, or in a climatic chamber or laboratory
- Outcomes:
  Individual effect of climate change–related environmental condition measured with wearables
  Validity and method comparison of wearables in extreme weather conditions

**Exclusion criteria**

- Publications:
  Nonhuman study population
  Reviews, editorials, or commentaries
- Wearable device:
  Not commercially available (eg, prototype or design study)
  Wearable with interventional function only (eg, cooling vest)
  Smartphone used as a wearable
  Wearable not implemented
- Climate change:
  Other environmental conditions
  Exposure to heat in the context of mining or firefighting
- Outcomes:
  Wearable (data) not specifically included in outcomes
  Environmental exposure or condition not included in outcomes
  Wearable only used to assess the effect of another intervention (eg, cooling)

Individual effects of climate change were limited to those resulting from exposure to weather extremes, as the topic would have been too broad otherwise [4]. As per the 2021-published Intergovernmental Panel on Climate Change report [3], we included exposure to heat and heat waves, heavy precipitation, floods, tropical cyclones, droughts, and wildfires. As heat and heat waves are often defined as extremes relative to the local climate (ie, daily minimum and maximum temperatures above the 95th or 99th percentile of the climatological record or a baseline period) [1,22], we relied on the definitions provided in the included studies. If the authors did not provide a definition, we used one of the following classifications, based on the available data in the screened articles:

- If data were available on wet bulb globe temperature (WBGT) [1,23] or the universal thermal climate index [24], we used >26 °C as a threshold.
- If data were available on ambient temperature and relative humidity, we calculated the heat stress index (HSI) [25] and used a threshold of >26 °C HSI.
- If data were available on ambient temperature, we used the average relative humidity at the study location (city or country) during the study period to calculate the HSI.

Studies on the effect of temperature on sleep were included even for lower ambient temperatures, as previous research has shown that small temperature changes already have adverse effects on sleep quality and duration [26] because humans only have a minimal ability to thermoregulate in rapid eye movement sleep phases [27]. We also included studies that reported on indoor heat exposure in climatic chambers or laboratories. We excluded studies on heat exposure during firefighting and mining, as we considered them job-specific and they predominantly assessed the microclimate inside the protective gear [28].

In case no full text was available or information on the wearables was missing, the authors were contacted 3 times before exclusion.

### Search Strategy and Information Sources

The full search was conducted on September 1, 2021, by 1 reviewer (MK) in 6 electronic databases: PubMed (MEDLINE), Scopus, CINAHL (EBSCOhost), IEEE Xplore, Ovid (MEDLINE[R]), and Web of Science. Gray literature was searched with Google Scholar, and the first 1000 search results were included [29]. We manually searched references of relevant included and excluded articles for further sources of evidence.

We followed the Population/Patients, Intervention, Comparison, and Outcome (PICO) framework to compile the search strategy. Population (P) included study participants wearing a wearable. Intervention (I) included exposure to climate change–induced weather extremes. No comparison (C) was required. Outcomes (O) included psychological and physiological health parameters or exposure measurable with wearables. Accordingly, the databases were searched using a search string including synonyms and medical subject headings terms for these concepts. Search strings were adapted to the specific requirements of each database (see Multimedia Appendix 2 for the full search strings). We applied a search filter for publications after January 1, 2010.

### Study Selection

The search results were imported into the literature reference management system EndNote 20 (Clarivate Analytics) and then imported into the systematic review management software Covidence (Veritas Health Innovation) where duplicates were removed automatically as well as manually. We screened titles and abstracts, as well as full texts, with application of the inclusion and exclusion criteria (see Textbox 1 for a full list of criteria). Subsequently, we extracted data from the included literature. The screening process was piloted prior with a sample of 20 articles. The literature was screened by 2 independent reviewers (MK and IM). Any disagreements were resolved by consensus between the 2 reviewers (MK and IM) and an independent researcher (SB).

### Data Extraction

A data-charting form was developed using the Covidence software template and piloted on 3 articles; data were charted by the 2 reviewers independently, and any disagreements were mutually resolved. The following data categories were extracted and synthesized [17,18]: title, author, year, country of study, objectives of study, demographics of the study population, sample size, methods, intervention type, outcomes, and key findings related to the scoping review question. In addition, the following items were extracted: wearable models, measured parameters with wearable, study setting, climate change–related environmental conditions including the measurement method, and methods used for data analysis or correlation.

### Synthesis of Results

The characteristics of the included studies and the study populations were summarized using Microsoft Excel (version 2206; Microsoft Corporation), and the study outcomes were narratively synthesized. The purpose of the use of the wearables was identified according to three categories: (1) validity and comparison in extreme conditions, (2) measuring individual exposure, or (3) measuring direct health effects.

## Results

### Overview

**Figure 1:**
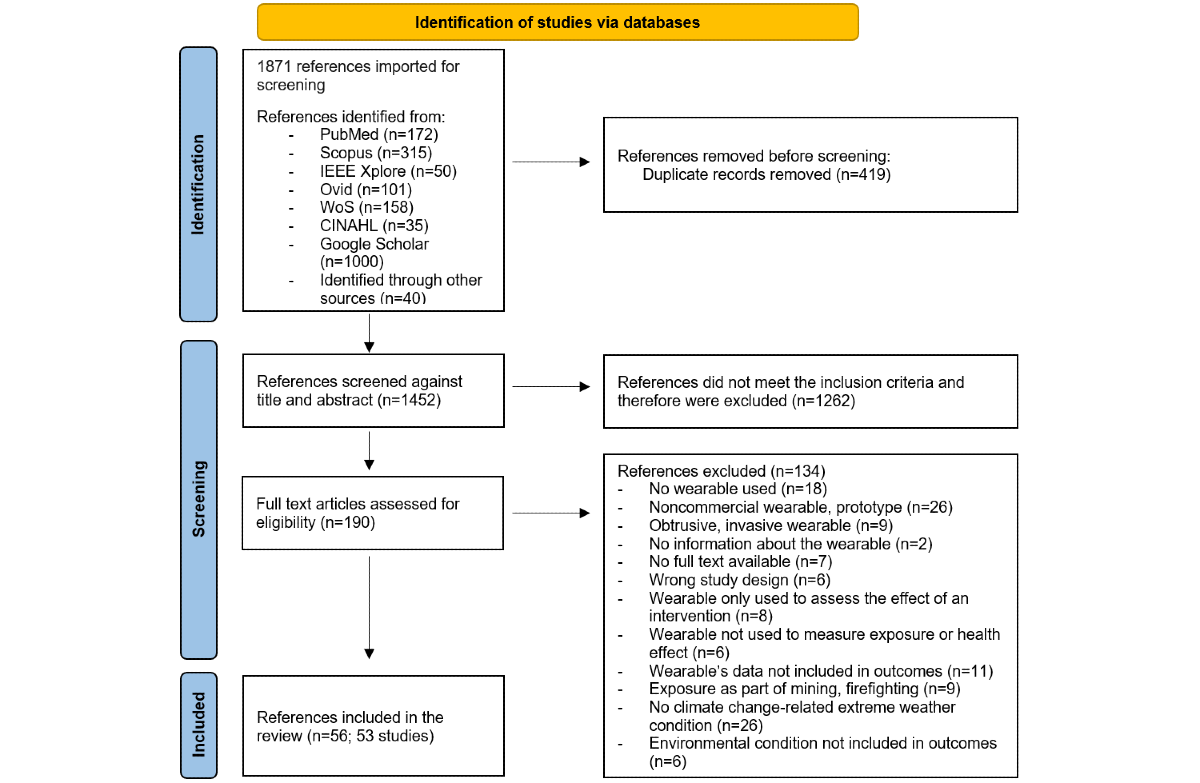
PRISMA (Preferred Reporting Items for Systematic Reviews and Meta-Analyses) flowchart.

The initial search yielded 1831 results, and 40 references were added after a manual search. We removed 419 duplicates and screened the titles and abstracts of the remaining 1452 nonduplicates. From a total of 190 screened full-text articles (186 studies), we included 53 studies (56 articles; see Figure 1 for the PRISMA [Preferred Reporting Items for Systematic Reviews and Meta-Analyses] flow diagram) including 1 preprint article [30]. For the conducting of this scoping review the preprint article was used and is therefore cited throughout the manuscript instead of the accepted article [31] that was published after our last search and data extraction process.

### Study Characteristics

**Figure 2:**
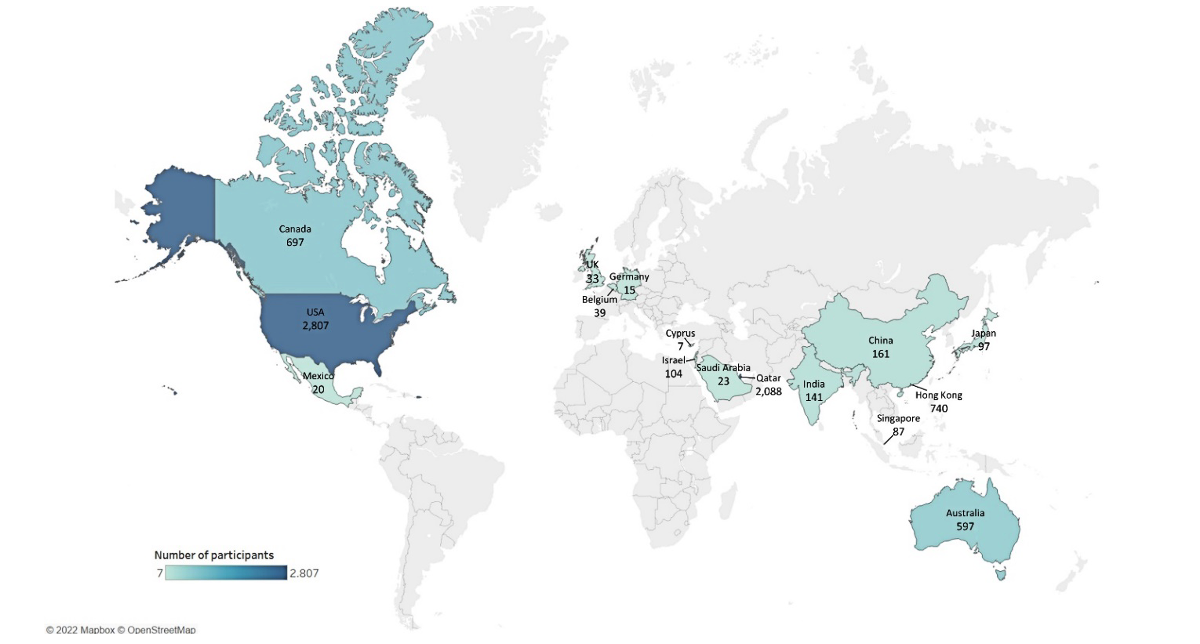
Map of study locations (countries). Minor et al [31] mentioned 68 countries across all continents (except Antarctica) but did not further specify, so they were not included in the map.

In total, we included a study population of 55,284 participants in this review (the characteristics of the included studies are summarized in Figure 2 and Table 1). Overall, there have been an increasing number of publications using wearables in the context of climate change and health research since 2010 (Table 2). The included studies were mostly observational (35/53, 66%) and crossover studies (21/53, 40%). Most studies were conducted in countries classified by the World Bank in 2022 [32] as upper–middle-income (5/53, 9%) and high-income countries (47/53, 87%), especially with more than half of the total studies conducted in North America (31/53, 58%). A few studies (4/53, 8%) included lower–middle-income countries. Most studies were conducted in urban settings (25/53, 47%) or in a climatic chamber (19/53, 36%), with a short study duration of up to 1 week (16/53, 30%) or up to 5 cross-sectional data collection points (17/53, 32%).

The median number of participants per study was 39 (range 6-47,628), comprising an average of 67% of male participants (Table 3 shows the demographics of the study population). In total, of the 53 studies, 15 (28%) studies focused solely on male participants versus 3 (6%) studies that only included female participants. A few studies (3/53, 6%) specifically included nonhealthy participants. Most study populations consisted of outdoor workers (14/53, 26%), including farm workers, construction workers, traffic police officers, or other workers, as well as the general population (11/53, 21%) or university members (students and staff; 7/53, 13%). Of the 53 studies, 2 (4%) studies included older adults and 4 (8%) studies included children. In addition, the study populations of individuals in the military, athletes, and homeless individuals were each represented in 2% (1/53) of studies.

**Table 1:** Study characteristics.

Study characteristics				Studies (N=53), n (%)		Participants (N=55,284), n (%)
**Regions and countries^a^**						
	**North America**			30 (56.6)		3524 (6.4)
		United States	24 (45.3)		2807 (5.1)	
		Canada	5 (9.4)		697 (1.3)	
		Mexico	1 (1.9)		20 (0)	
	**Asia**			11 (20.8)		1226 (2.2)
		Hong Kong	1 (1.9)		740 (1.3)	
		China	3 (5.7)		161 (0.3)	
		India	3 (5.7)		141 (0.3)	
		Japan	2 (3.8)		97 (0.2)	
		Singapore	2 (3.8)		87 (0.2)	
	**Europe**			5 (9.4)		94 (0.2)
		Belgium	1 (1.9)		39 (0.1)	
		United Kingdom	2 (3.8)		33 (0.1)	
		Germany	1 (1.9)		15 (0)	
		Cyprus	1 (1.9)		7 (0)	
	**Oceania**			4 (7.5)		597 (1.1)
		Australia	4 (7.5)		597 (1.1)	
	**Middle East**			3 (5.7)		2192 (4)
		Qatar	1 (1.9)		2088 (3. 8)	
		Israel	1 (1.9)		104 (0.2)	
		Saudi Arabia	1 (1.9)		23 (0)	
	South America			0 (0.0)		0 (0)
	Africa			0 (0.0)		0 (0)
	Countries not specified (68 countries: 42 high-income countries; 17 upper–middle-income countries; 9 lower–middle-income countries)			1 (1.9)		47,628 (86.2)
**Study setting^a,b^**						
	**Urban**			31 (58.5)		—^c^
		Outdoor	9 (17.0)		—	
		Indoor	5 (9.4)		—	
		Indoor and outdoor	17 (32.1)		—	
	**Rural**			11 (20.8)		—
		Outdoor	7 (13.2)		—	
		Indoor	1 (1.9)		—	
		Indoor and outdoor	3 (5.7)		—	
	Climatic chamber or laboratory			19 (35.8)		—
**Study duration**						
	**Cross-sectional data collection points (up to 4 hours each)**					
		≤5 data collection points	17 (32.1)		496 (0.9)	
		≥6 and ≤10 data collection points	3 (5.7)		174 (0.3)	
		≥11 and ≤50 data collection points	3 (5.7)		116 (0.2)	
	**Continuous monitoring (at least 1 [work] day)**					
		≤7 days	16 (30.2)		3614 (6.5)	
		≤1 months	6 (11.3)		171 (0.3)	
		≤6 months	5 (9.4)		542 (1)	
		≤2 years	3 (5.7)		50,171 (90.8)	
**Study design**						
	Experimental crossover study^d^			7 (13.2)		210 (0.4)
	Prospective cohort study			20 (37.7)		49,690 (89.9)
	Retrospective cohort study			1 (1.9)		104 (0.2)
	Prospective observational crossover study^d^			14 (26.4)		5017 (9.1)
	Method comparison or evaluation study			11 (20.8)		263 (0.5)

^a^Multiple characteristics may apply per study.

^b^Information for study settings is not available for all study participants and therefore not summarized here as the number of participants per study setting.

^c^Not available.

^d^Each participant serves as their own control or comparison.

**Table 2:** Years of publication.

Year of publication	Included publications (N=56), n (%)
2013	3 (5)
2014	6 (11)
2015	6 (11)
2016	5 (9)
2017	5 (9)
2018	6 (11)
2019	8 (14)
2020	11 (20)
2021 (until September 1)	6 (11)

**Table 3:** Demographics of included studies.

Study	Participants monitored with wearables, n	Study population	Sex (male), %	Age (years)	Ethnicity, %
Al-Bouwarthan et al [33], 2020	23	Construction worker	100	Mean 42.7 (SD 8.8)	—^a^
Al-Mohannadi et al [34], 2016	2088	General population	67	Range 18-65	—
Al Sayed et al [35], 2017	12	Male	100	Mean 24.8 (SD 3.8)	—
Bailey et al [36], 2019	38	University member	50	Group 1: mean 32.6 (SD 13); group 2: mean 21.5 (SD 3)	92% White
Benita et al [37], 2020; Benita and Tuncer [38], 2019	10	University student; female	0	Mean 22.8 (SD 1.5)	—
Benjamin et al [39], 2020	19	Athlete; female	0	Mean 20.6 (SD 1.4)	—
Bernhard et al [40], 2015	81	Outdoor worker or general population	35	Mean 52 (rural), 50.5 (urban), and 44.5 (outdoor worker)	93% Black or African American
Cedeño Laurent et al [41], 2018	44	University student; healthy	51	Mean 20.2 (SD 1.8)	40% White
Cheong et al [42], 2020	9	Older adult	22	Range 65-87	67% White, 11% Black, 11% Hispanic or Latino, and 11% other
Cuddy et al [43], 2013	56	Male	100	Mean 22 (SD 3)	—
Culp and Tonelli [44], 2019	20	Farm worker; male	100	Range 18-65	100% Hispanic
Edwards et al [45], 2015	372	Children (age 3 years at recruitment); healthy	52	Mean 3.4 (SD 0.3)	22% Black or African American
Hamatani et al [46], 2017	13	General population	92	—	—
Hass and Ellis [47], 2019	45	General population	37	Range 18-≥65	64% White and 11% Black or African American
Hondula et al [48], 2020	84	General population	—	—	—
Ioannou et al [49], 2017	7	Farm worker; healthy	71	Male: mean 39 (SD 10.8); female: mean 39.5 (SD 13.4)	—
Jehn et al [50], 2014	15	Clinically stable NYHA II-IV^b^ patients with PAH^c^	60	Mean 66.7 (SD 5.2)	—
Kakamu et al [51], 2021	84	Construction worker	100	Mean 48.4 (SD 14)	—
Ketko et al [52], 2014	104	Military; male	100	Range 18-21	—
Kim et al [53], 2013	12	Male	100	Mean 25.5 (SD 4.1)	—
Kuras et al [54], 2015	23	General population	39	Range 25-79	74% White and 26% Black or African American
Lam et al [55], 2021	145	University student (first-year student)	34	Mean 18.1 (range 17-21)	—
Larose et al [56], 2014	60	Male; healthy	100	Mean 45.4 (range 20-70)	—
Lewis et al [57], 2016	1095	Children aged 9-11 years	43	Mean 10.6 (SD 0.4)	—
Li et al [58], 2020	10	Construction worker; healthy; male	100	Mean 39.4 (SD 3.6)	—
Lisman et al [59], 2014	46	Military or university community member; healthy or previous exertional heat stroke	74	Mean 29.7 (SD 5.9)	—
Longo et al [60], 2017	20	Homeless individual or university student	75	Range 18-60	—
Lundgren et al [61], 2014	77	Outdoor worker	86	—	—
MacLean et al [62], 2020	12	Male; healthy	100	Mean 24.2 (SD 3.7)	—
Minor et al [30], 2020	47,628	General population	69	Age distribution: 19-25, 6%; 25-65, 91%; ≥65, 3%	—
Mitchell et al [63], 2018	587	Farm worker	66	Mean 38.6	98% Latino
Nazarian et al [64], 2021	77	General population	52	Range 18-48	100% Asian
Notley et al [65], 2021	50	Young (18-30) and healthy or older (50-70) and healthy; older and T2D^d^ or HTN^e^	100	Mean 50 (SD 17); mean per group: 22 (young), 58 (older), 60 (T2D), and 61 (HTN)	—
Ojha et al [66], 2020	10	University student	70	—	—
Pancardo et al [67], 2015	20	Outdoor worker; healthy	55	Mean 28.6 (range 22-51)	—
Quante et al [68], 2017	669	Adolescents aged 12-14 years	49	Mean 12.9 (SD 0.6)	68% White, 14% Black, 3% Hispanic, 3% Asian, and 13% Other
Raval et al [69], 2018	16	Traffic police worker	100	Range 19-57	—
Ravanelli et al [70], 2016; Ravanelli et al [71], 2015	8	Male; healthy	100	Mean 24 (SD 3)	—
Relf et al [72], 2018	14	Female; healthy	0	Mean 26 (SD 7)	—
Relf et al [73], 2020	19	General population; healthy	79	Mean 41 (SD 23)	—
Rosenthal et al [74], 2020	455	General population	42	—	—
Runkle et al [75], 2019; Sugg et al [76], 2018	35	Outdoor worker	100	Mean 39.2	74% White, 14% Black or African American, 9% Hispanic, and 2% American Indian or Alaska Native
Sahu et al [77], 2013	48	Farm worker	100	Range 25-34	—
Seo et al [78], 2016	12	Male; healthy	100	Group 1: mean 23 (SD 1); group 2: mean 23 (SD 2); group 3: mean 24 (SD 2)	—
Shakerian et al [79], 2021	18	University student	78	Female: mean 24 (SD 3.2); male: mean 24 (SD 2.8)	—
Shin et al [80], 2015	9	Young; healthy	67	Mean 23.3 (SD 4.1)	—
Suwei et al [81], 2019	51	Outdoor worker	35	Mean 42.9 (range 21-60)	96% African American
Uejio et al [82], 2018	50	Outdoor worker	92	Mean 44 (SD 11.1)	59% Black, 39% White, and 2% Hispanic
Van Hoye et al [83], 2014	39	University student; healthy	54	Mean 21.4 (SD 1.41)	—
Williams et al [84], 2019	51	Older adult	43	Mean 65.4	67% White
Xiong et al [85], 2020	48	General population	46	Mean 36 (SD 12)	—
Zheng et al [86], 2019	740	Adolescent or secondary school student	52	Mean 14.7 (SD 1.6)	100% Asian
Zhu et al [87], 2016	6	General population	50	Males: mean 27.3 (SD 2.5); female: mean 22.3 (SD 1.2)	—

^a^The respective information was missing in the article.

^b^NYHA II-IV: New York Heart Association Functional Classification for heart failure stage II-IV.

^c^PAH: pulmonary arterial hypertension.

^d^T2D: type 2 diabetes.

^e^HTN: hypertension.

### Wearable Devices

Most of the included studies used 1 (39/53, 74%) or 2 (12/53, 23%) wearables; a few studies (2/53, 4%) used ≥3 devices (study methods and objectives detailed in Table 4). The 70 wearables in the included studies were from 23 different companies overall with Polar Electro (16/53, 30%), Maxim Integrated (13/53, 25%), and Fitbit (5/53, 9%) providing the most frequently used wearables. The most commonly reported use for wearables was the measurement of HR (30/53, 57%), physical activity (15/53, 28%), or individually experienced temperature (IET; the air temperature surrounding the individuals; 14/53, 26%). Other parameters included sleep (duration, onset, wake time, etc), energy expenditure, skin temperature, electrodermal activity, local sweat rate, respiratory rate, or geoposition. Some wearables measured multiple parameters. The devices were mostly wristbands (25/70, 36%), chest straps (18/70, 25%), clipped to clothing or accessories (15/70, 21%), or directly taped to the skin (5/70, 7%). All included studies additionally used questionnaires and further health parameters (eg, blood pressure, weight, height, and urine samples).

**Table 4:** Study methods and objectives.

Methods and objectives				Studies (N=53), n (%)
**Number of wearables per study**				
	1		37 (74)	
	2		12 (23)	
	≥3		2 (4)	
**Wearable company (models)^a^**				
	Polar Electro (RCX3, H7, RS800XC, FT1, FT7, Team 2 [Pro], RS800, RS400, WearLink, Accurex Plus, A300, and M400)		16 (30)	
	Maxim Integrated (iButton Hygrochron and Thermochron)		13 (25)	
	Fitbit (Ionic, Charge 2, and Flex)		5 (9)	
	Medtronic (Zephyr BioHarness)		4 (8)	
	Philips Respironics (Actical and Actiwatch 2), Onset Corp (HOBO Pendant), and Empatica (E4)		3 (6; each)	
	Crossbridge Scientific (KuduSmart), Actigraph (GT3X and GT3X+), Intel (Basis Peak Watch), BodyMedia (SenseWear Pro 3), Sony (SmartBand Talk SWR30 and SWR12)		2 (4; each)	
	Omron Healthcare (HJ-720 ITC pedometer), STATSports (Viper Pod), Microsoft (Band), Garmin (Vivoactive HR), Aipermon (APM), Stayhealthy (RT3), GISupply (LW-360HR), Lifensense (Mambo 2), LASCAR (EL-USB-2-LCD+), Easylog (Easylog), PAL Technologies (activPAL and activPAL3C)		1 (2; each)	
**Measured parameter with wearable^a^**				
	Heart rate		30 (57)	
	Physical activity		15 (28)	
	Energy expenditure		8 (15)	
	Skin temperature		12 (23)	
	Electrodermal activity		5 (9)	
	Sleep (onset, offset duration, and efficiency)		7 (13)	
	Individually experienced temperature		14 (26)	
	Others (local sweat rate, respiratory rate, and GPS location)		7 (13)	
**Wear location of wearable^a^**				
	Wristband		25 (47)	
	Chest strap		18 (34)	
	Attached to clothing or accessories		15 (28)	
	Taped to the skin		5 (9)	
	Other: shirt, back strap, around upper arm, or not specified		8 (15)	
**Climate change–related extreme weather**				
	Heat		52 (98)	
	Wildfire		1 (2)	
**Measured environmental condition^a^**				
	Temperature		50 (94)	
	Relative humidity		40 (75)	
	Precipitation		7 (13)	
	Other (wind speed, wet bulb temperature, dry bulb temperature, dew point, mean radiant temperature, barometric pressure, visibility, CO_2_ concentration, and air quality)		22 (42)	
**Measurement location or data source for environmental condition^a^**				
	Nearest weather station		20 (38)	
	Sensors placed on study site		18 (34)	
	Climatic chamber or laboratory		18 (34)	
	Locally installed weather station		4 (8)	
	Smartphone sensor		2 (4)	
	Satellite data		2 (4)	
**Heat stress measure^a^**				
	Wet bulb globe temperature		14 (26)	
	Heat stress index		5 (9)	
	Humidex		2 (4)	
	Others (universal thermal climate index, heating or cooling degrees, heat stroke index, heat stress days, heat stress level estimation, heat balance equation, extreme heat degree minutes, and physiological equivalent temperature)		1 (2; each)	
	None		27 (51)	
**Method of analysis (statistical test)^a^**				
	Regression (linear, logistic, and Cox)		16 (30)	
	Linear mixed effect model		16 (30)	
	Time-series analysis		1 (2)	
	*t* test (2-tailed or 1-tailed)		21 (30)	
	Correlation (Pearson, Spearman, etc)		13 (25)	
	ANOVA (one-way, repeated measures, and mixed design)		14 (26)	
	MANOVA		1 (2)	
	Nonparametric test (Wilcoxon *U* test and Kruskal-Wallis test)		7 (13)	
	Chi-square and Fisher Exact Test		4 (8)	
	Bland Altman plot		5 (9)	
	Spatial correlation		1 (2)	
	Cohen kappa		1 (2)	
	Descriptive analysis only		5 (9)	
**Study objectives and use of wearables^a^**				
	**Studies measuring the correlation of wearables’ data and environmental conditions**			
		Effect of heat on sleep	7 (13)	
		Effect of heat on physical activity	7 (13)	
		Effect of heat on heart rate	10 (19)	
		Other physical responses to heat	6 (11)	
		Occupational heat stress	8 (15)	
		Effect of wildfires on physical activity	1 (2)	
	Studies measuring the individual experienced temperature and comparing it to local or area measurements		10 (19)	
	Studies assessing the validity and applicability of wearables for their use in extreme weather		14 (26)	

^a^Multiple characteristics may apply per study.

### Weather or Climate Data

The primary focus was on the use of wearables to measure physiological responses to heat exposure (52/53, 98%). Of the 53 studies, 1 (2%) study assessed the impact of forest fires on individual activity, and 5 (9%) measured the effect of precipitation on activity in addition to heat. The weather or climate conditions were predominantly assessed using data from the nearest weather station (20/53, 38%), sensors placed on the study site (18/53, 34%), or measured in a climatic chamber or laboratory (18/53, 34%). Others accessed weather data from locally installed weather stations, built-in sensors of participants’ smartphones, or satellite data. Besides the primarily focused measurements of temperature, precipitation, and relative humidity, 49% (26/53) of the included studies calculated different heat stress indices (eg, WBGT, HSI, or universal thermal climate index).

### Statistical Analysis

The methods of statistical analysis of wearables’ data and correlation to climate or weather data were primarily regression, linear mixed effect models, correlation, ANOVA, and 1- or 2-tailed *t* tests (Table 5). Linear regression models or linear mixed effect models, for example, were often used to correlate IETs and area-level temperature data [35,40,42,48,75], but *t* tests were also used for the comparison between both methods [47,54]. Data sources differed between group-level data and participant-level data [42,54]. The associations of heat exposure and wearables-measured parameters were mostly examined with linear mixed effect models or different regression models (linear, logistic, or Cox), adjusted for age, sex, and education [33,34,39,41,42,45,63,65,68,84-86]. For the comparison of the effect of heat between groups with different characteristics such as sex or age and for the comparison of heat-stress and non–heat-stress days, *t* tests, Chi-square tests, and ANOVAs were used [35,37,43,50,55-57,59,61,63,65,84]. Studies that compared wearables measurements with standard devices applied; in addition to *t* tests and ANOVAs, different correlation coefficients and Bland Altman plots for the appraisal of disagreement [36,72,73,78,83]. Other analysis methods were also used. One study [37,38] spatially correlated different urban environmental exposures and body responses during a 10-minute walk in Singapore. Pattern recognition and parametric tests were used to identify stress hot spots on this walk, and 4 machine learning models were trained to test the predictive power of the immediate environment. Another study used machine learning models for heat strain assessment [79].

**Table 5:** Study findings regarding the associations of demographic characteristics and heat exposure or physical response.

Finding			Adverse effects on sleep		HR^a^ increase		Decrease in physical activity		Skin temperature increase		Occupational heat stress		Higher IET^b^
**Age (years)**													
	Positive association	[30]		[56]		[34]		—^c^		[63]		[40]	
	Null association	—		[65]		—		—		—		—	
**Sex (female)**													
	Positive association	[30]		[59]		[34]		—		[61,63]		—	
**BMI or body fat percentage**													
	Positive association	—		[59]		—		[44]		[63,75,76]		[40]	
**Education**													
	Positive association	—		—		—		—		[75,76]		—	
	Negative association	—		—		—		—		—		[47]	
**Income**													
	Negative association	—		—		—		—		—		[40,47,88]	
**Homelessness**													
	Positive association	—		—		—		—		—		[60]	
**Health status (hypertension and type 2 diabetes)**													
	Null association	—		[65]		—		—		—		—	
**Lower-income country**													
	Positive association	[30]		—		—		—		—		—	
**From the Eastern Mediterranean region**													
	Positive association	—		—		[34]		—		—		—	

^a^HR: heart rate.

^b^IET: individually experienced temperature.

^c^No findings regarding an association were stated in the included studies.

### Study Outcomes and Findings Regarding the Use of Wearables in Extreme Weather Conditions

#### Overview

**Figure 3:**
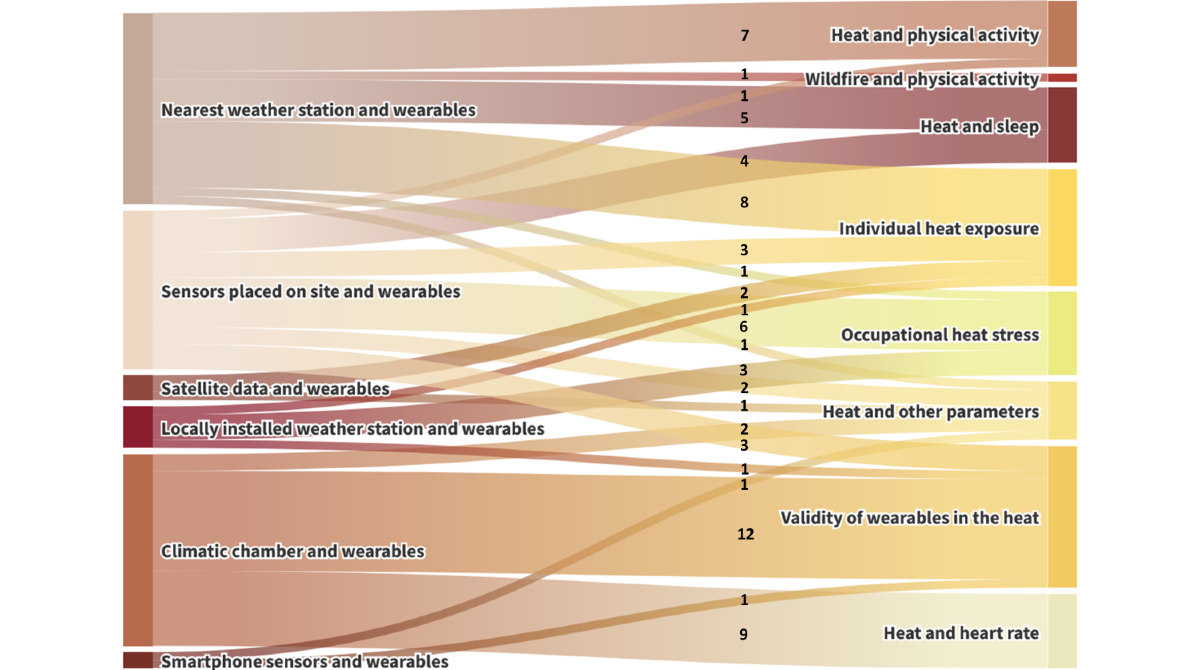
The Sankey diagram shows the data collection methods that were used for each study outcome. The methods are displayed on the left (weather or climate measurement method and wearable) and connected to the respective study outcome shown on the right. The numbers show the number of studies that are represented by each link. One study might have more than one study outcome and therefore could be represented in multiple strings.

We categorized the studies according to the use of wearables in extreme weather conditions (Table 4). An overview of the data collection methods for each category is shown in Figure 3. Table 5 displays the reported associations of participants’ demographic characteristics and individual exposure or physiological responses to heat. The study findings are summarized in the subsequent sections (see Multimedia Appendix 3 for a detailed compilation).

#### Correlation of Wearables’ Data and Extreme Weather

##### Effect of Heat on Sleep

Of the 53 studies, 7 (13%) studies [30,41,68,80,84,85,87] examined the effect of bedroom environmental conditions on sleep parameters (efficiency, rapid eye movement sleep, duration, sleep onset latency, and sleep disruptions) and all 7 studies found a negative correlation between higher ambient temperature and sleep in their study cohorts. One large-scale study [30] found significantly larger negative effects of heat on sleep duration for residents from lower-income countries, older adults, and females, with no evidence of short-term acclimatization. In contrast, another study [41] found evidence of short-term heat acclimatization in a cohort of young adults.

##### Effect of Heat on Physical Activity

A total of 13% (7/53) of studies [34,39,45,50,57,68,86] examined the effect of heat on physical activity (mostly measured in the form of steps), and most (6/7, 86%) studies [34,39,45,50,57,68] found a negative correlation in the general population, children, female soccer players, and patients with pulmonary arterial hypertension and clinically stable heart insufficiency (New York Heart Association Classification Stage II-IV). In contrast, one study [86] found a significant positive correlation for temperatures between 13 °C and 31 °C in a cohort of children on weekend days. One study [34] found that the decrease in physical activity was greater with age in female participants and participants from the Eastern Mediterranean region; Five studies [34,45,57,68,86] examined the effects of precipitation and heat on physical activity, and all (5/5, 100%) found a negative correlation between precipitation and physical activity. Four of these studies [45,57,68,86] observed this effect in cohorts of children.

##### Effect of Heat on HR

Of the 53 studies, 10 (19%) studies [35,43,52,56,58,59,65, 66,70,71,83] examined the effect of heat on HR, and most (7/10, 70%) observed increasing HR in hotter and more humid conditions, especially in older adults. Four studies [35,56,66,83] found no significant effect of hot and humid conditions on HR during exercise among young adults, whereas 1 study [58] found no significant effect in middle-aged participants. In contrast, 1 study [65] found no significant difference in HR response to exercise under heat exposure for different age groups or participants with hypertension or type 2 diabetes. Two studies [43,52] conducting heat exposure tests measured significantly higher, steadily increasing HR for participants classified as “at risk” or “heat intolerant.” One study [59] found that BMI, percent body fat, sex, and maximal oxygen uptake were associated with elevated HR during heat tolerance testing. To mitigate these heat effects, 1 study [70,71] found that placing an electric fan 1 m in front of the participants could significantly delay HR increase in hot and humid conditions.

##### Other Physiological (and Psychological) Responses to Heat

Of the 53 studies, 6 (11%) studies [37,38,42,55,66,73,83] examined further body responses to heat exposure, including increasing skin temperature, electrodermal activity, skin conductance response, and energy expenditure (during high-intensity exercise). One study [37,38] discovered stress hotspots during a route through the city, which may be explained by changes in the immediate environment, such as the transition from a park to a residential area or an exposed area without shade. Another study [42] found a correlation among higher HR, near-body temperature, and outside temperature when participants reported mild anxiety.

Two studies observed signs of short-term heat acclimatization over 9 to 10 days in the form of increasing local sweat rate [73] and decreasing HRs and metabolic rates for both local and nonlocal students. The differences between both groups (higher values for nonlocal students) were assimilated in the second week [55].

##### Occupational Heat Stress

Of the 53 studies, 8 (15%) studies [33,44,49,51,61,63,75-77] used wearables to investigate the physiological effects of occupational heat stress on outdoor workers, including construction, farm, and ground management workers. Six studies [44,49,61,63,75-77] found an association between occupational heat exposure and physiological responses, including increasing HR [44,75-77], metabolic rate [61], skin temperature [44,49], and decreasing physical activity [63]. One study [33] found that WBGT and heat stress exposure were stronger predictors of cardiovascular strain (measured as HR reserve) than energy expenditure during construction work. Five studies found associations between demographic characteristics and physical responses to occupational heat stress, including the female sex (2/8, 25%) [61,63], older age (1/8, 13%) [63], higher BMI (2/8, 25%) [44,75,76], and education (1/8, 13%) [75,76]. Furthermore, of the 8 studies, in 1 study [75,76], the perception of heat as an occupational hazard and officially issued heat alerts were associated with a lower heat strain risk. Two studies [33,61] observed that occupational heat stress exposure frequently reached critical conditions.

##### Effect of Air Quality During Wildfires on Physical Activity

Of the 53 studies, 1 (2%) study [74] found a statistically significant reduction in daily step counts with progressively worse air quality during wildfires, with an 18% reduction in daily step count when the air quality index exceeded 200 (considered very unhealthy, and public health warnings were typically issued) compared with less than 100 (considered good air quality).

#### Individual Heat Exposure and Comparison to Area-Level Measurements

Of the 53 studies, 10 (19%) studies compared individual heat exposure measured with wearables to area-level measurements [36,40,42,47,48,54,60,69,81,82]. Individual heat exposure was measured in the form of IET (the air temperature surrounding the individuals).

Three studies [42,48,54] found high heterogeneity in IETs, indicating interindividual differences in time spent outdoors, 2 of which [42,48] found little to no correlation between temperature measured at the nearest weather station and IET while participants spent their time indoors and outdoors. Two studies [36,54] found different associations between IETs and weather station measurements between daytime and nighttime. Three studies [69,81,82] observed higher IETs when compared with measurements from the nearest weather station or the locally installed weather station for outdoor workers [69,81,82]; individual exposure frequently exceeded the recommended values. In contrast, 4 studies [40,47,48,54] found that average temperature measurements from the nearest weather station were higher than average daily IETs, including heat wave periods compared with non–heat wave periods (2/10, 20%) [47,54]. Four studies [40,47,48,60] found associations between demographic characteristics and IET, including a negative association of income (3/10, 30%) [40,47,48], education (1/10, 10%) [47], homelessness (1/10, 10%) [60], urban environment (1/10, 10%) [40], and higher body fat percentage (1/10, 10%) [40] with heat exposure (especially indoors and during the night), whereas older study participants experienced higher strain due to nighttime exposure compared with the comparison groups (1/10, 10%) [40].

#### Validity and Reliability of Wearables in Extreme Weather

Of the 53 studies, a total of 14 (26%) studies [35,36,43,46,52,53,62,64,67,72,73,78-80,83] evaluated and compared different wearables and their validity and reliability in the context of heat exposures.

Seven different wearables were compared with gold-standard methods for measuring various physiological parameters during heat exposure and under different levels of physical activity. Five studies found no significant differences in measurements between wearables and gold-standard devices, including the Polar chest strap [35], Hexoskin shirt [35], and Zephyr BioHarness [53] for HR measurements, the SenseWear Pro 3 and Actiwatch 2 wearables [80] for the measurement of sleep, 2 different SenseWear Algorithms for the estimation of energy expenditure during low-intensity activity [83], and the KuduSmart [72,73] for local sweat rate measurements. Two studies [36,62] compared wearables and their placements using standard measurements. One study [62] concluded that single-location skin temperature measurements at the chest, scapula, and thigh with iButton wearables taped to the skin were the only positions to agree with mean skin temperature (standard method) under all conditions and that wearables outperformed the infrared device. The other study [36] found no significant difference between sensor types (iButton Thermochron and Hygrochron, HOBO Pendant) and placements (on shirt collar, shoe, or backpack) for the measurement of IET but the correlation between sensors was the lowest during high-intensity activities.

Three studies (3/14, 21%) [46,64,78] compared different methods of using wearables data for core temperature estimation to measured values and found good overall results using HR [78]; HR and skin temperature [46]; and HR, skin temperature, and near-body temperature [64]. Four studies [43,52,67,79] evaluated different methods to use wearables data (HR or cardiac cost, electrodermal activity, and skin temperature) to assess heat strain and all found high sensitivity or accuracy. Two of these studies paired wearables data with skin temperature or rectal and core temperature measurements for assessment.

## Discussion

### Principal Findings

Overall, the included studies revealed a diverse spectrum of wearable devices, with a particular emphasis on physical responses to heat. Heat was found to adversely affect sleep, physical activity, occupational stress exposure, and other physical and psychological parameters. Air quality during wildfires was another weather extreme examined in 1 study and was found to negatively affect physical activity. A comparison of individual exposures against weather station area-level measurements showed high differences. Wearables were found to provide valid and reliable metrics for assessing physiological responses in extreme weather conditions. We identified a slight increase in the number of scientific publications in recent years.

### Study Settings

The vast majority of studies were conducted in upper–middle-income and high-income countries, as has been reported in prior publications [2]. Half of the study participants in the included studies were from North America, even though sub-Saharan Africa, South and Central America, and parts of Asia are projected to be the regions most affected by climate change [12]. Different obstacles to the use of wearables in low-income countries could be the reason for this finding. From the participants’ perspective, acceptability of and adherence to the use of wearables could be affected by skepticism [89] or fear of theft and loss of the device [90], since these populations are often not as exposed to these technologies as study populations from high-income countries [91,92]. From the researchers’ perspective, another obstacle could be the lack of smartphones that are needed for the connection of most wearables and lack of high-speed internet connections [90,91] and therefore higher study costs. Some studies [30,74] have included participants who already owned wearables, which translates into higher acceptability by potential study participants. However, this approach is hardly possible in populations where wearables are not commonly used [92]. A few studies are ongoing in low-income countries, among others is a study exploring the feasibility of consumer-grade wearable devices in Burkina Faso and Kenya [93]. However, not only wearables data present an obstacle for studies in low-resource contexts but also weather and climate data are not as widely available with less granular spatial distribution of weather stations, especially in remote regions [94]. Using other systems to conduct weather data, such as small, portable sensor systems, has been found to be a possibility for low-income countries [95], and within the framework of some studies, sensors or weather stations were installed in the study region [36,49,63,75,76]. Many of the included studies were conducted in laboratory settings or ran over a short study duration with few participants. Laboratory settings do not necessarily reflect real life and cause low ecological validity. We found only a few studies [30,34,57] that conducted large-scale studies in real-life settings of the participants. By contacting wearable users and collecting their data [30,96], large-scale studies over long periods with population sizes as big as half a million can be conducted quite easily. When the conducted data are correlated with available weather data, important insights into the continuous and long-term health effects of climate change can be gained.

### Study Populations

The study populations of the included studies primarily comprised healthy participants and only few that included cohorts with vulnerable populations, such as patients with diabetes or heart insufficiency [50,65]. Wearables have previously been used to assess health in cohorts of patients with chronic diseases, such as cardiovascular diseases [97], and could provide an opportunity to better assess vulnerability to climate change in patients with pre-existing conditions. Different age groups and the association between age and vulnerability to heat have been examined in some studies, mainly showing a higher negative impact of heat on sleep, physical activity, and HR for older participants, as well as higher exposure to nighttime heat. In addition, other demographic characteristics, such as higher BMI and lower income, were associated with heat exposure and larger health effects in some studies. These findings on immediate and short-term health effects are consistent with those of other studies that assessed severe health outcomes, such as hospitalization rates [5]. However, not all studies found similar results; for example, Notley et al [65] found no significant differences between age groups or between healthy and nonhealthy participants. Overall, more research is needed to understand the causal relationship between different population characteristics and the health effects of climate change or climate change–induced weather extremes.

### Wearables

The range of included wearables and measurements was broad; however, most studies only used HR, temperature, and accelerometry data. In addition, the validity of various wearables was confirmed to be high in extreme conditions, such as heat, including heat strain assessment, and to be an accepted, noninvasive method for evaluating core temperature.

We also found that many studies used iButtons from Maxim Integrated for ambient and skin temperature measurements; some studies taped the devices to the skin with medical tape. However, we could not find any information from the manufacturer on whether their devices are suitable for measuring skin temperature in this manner.

### Weather or Climate Data

Our findings indicate that most studies relied on weather station data, with weather stations frequently located outside cities (eg, airports), and that most studies provided no information on the distance between the weather stations and the study population. However, most studies comparing IETs and area-level measurements from the nearest weather station have shown that they often do not accurately represent individual heat exposure. Future studies should consider individual-level measurements for a more exact heat prediction that captures the effects of time spent indoors and heat adaptation measures such as air conditioning. Almost all of the research has addressed one climate change–related weather extreme: heat. Although heat poses one of the most immediate health threats of climate change [1], other weather extremes and their health effects should not be neglected.

### Health Effects of Extreme Weather

Most of the included studies showed an association of weather extremes with adverse health outcomes, particularly for heat. The adverse health effects of heat included less sleep (quality), less physical activity, increased HR, and higher skin temperature. A few studies have also observed changes in physical response to heat, indicating short-term heat adaptation [41,55]. The heat effects are consistent with those of prior studies that did not use wearables [5,26,98-100]. Studies examining occupational heat stress using wearables found physical effects similar to those mentioned above for other study populations, including cardiac strain and decreasing physical activity [44,49,61,63,75-77]. The physical effects of occupational heat stress are extensive and may impose a significant economic burden owing to the decreased working capacity [101]. The IET by outdoor workers is often higher than area-level measurements and frequently reaches critical thermal conditions during work [33,61], which is important to consider for the assessment of occupational safety.

### Individual Heat Exposure

The included studies assessed individual heat exposure with IET, which mostly did not align with area-level measurements and showed high interindividual variances. Sociodemographic factors such as age, income, and education were found to be associated with higher heat exposure, potentially due to a lack of access to cooling methods, such as air conditioning, which have been shown to successfully mitigate the adverse effects of heat [42]. Lower IETs were found during heat waves when heat warnings were made publicly available compared with non–heat wave periods, emphasizing the importance of publicly available information as a means of mitigating individual heat adaptation strategies. In some studies [54,84], indoor temperatures during the night were higher than outdoor temperatures, even with access to air conditioning. This could be explained by the slower cooling rates indoors owing to heat storage in buildings [102]. However, it is important to note that body heat might affect IET measurements by wearables worn on the body or clothes. This aspect was not considered in the included articles.

### Limitations

One of the limitations of this review is the exclusion of prototype wearables. Even though studies with prototypes are hardly reproducible, they (especially large-scale studies) may provide valuable insights into the direct health effects of climate change that have not been considered in this review. In addition, we had to exclude many studies because they used invasive or obtrusive wearables. These devices may affect the participants’ compliance and make conducting a study outside the laboratory challenging. Second, with the limitation of climate change–related conditions to extreme weather events, we excluded other effects of climate change as well as moderate effects. Other studies and reviews have examined the effects of seasons and moderate weather conditions on health [103,104]. Furthermore, the definitions of extreme weather conditions in our review, especially heat, were not consistent; therefore, comparisons between the study outcomes must be considered cautiously. Third, we excluded studies that did not use wearables to measure the effects of extreme weather, but the effects of interventions, such as studies comparing the effects of fan use and air conditioning. Studies that assessed interventions against heat stress [105,106] could provide important insights into how to best prevent the adverse health effects of climate change.

### Conclusions

We found a broad range of wearables to be used in the context of climate change and health research. The validity of many wearables compared with standard devices or methods is high, even in extreme heat. The included studies found that the effects of extreme weather conditions on health can be examined and correlated with wearable data. They showed that heat has adverse effects on wearables-measured variables such as HR, physical activity, and sleep. The findings have underlined individual factors to be associated with higher vulnerability. Furthermore, wearables have been demonstrated to be a suitable tool for assessing individual heat exposure. This could be especially valuable if other meteorological data are not available or during exposure times with large disparities between individual- and area-level measures, such as heat waves or at night. We have identified gaps in the research regarding the use of wearables in low-income contexts and for long-term observations in large-scale studies. For further research, wearables may be a valuable method to generate insights and data at the individual level to better understand the impact of climate change on health, including moderate and short-term effects. As a next step, wearables could be used for the evaluation of adaptation measures.

## Appendix

Multimedia Appendix 1 PRISMA-ScR (Preferred Reporting Items for Systematic Reviews and Meta-Analyses extension for Scoping Reviews) checklist.

Multimedia Appendix 2 Search strings for each database.

Multimedia Appendix 3 Information about the wearables and weather or climate measures for each study.

## Abbreviations

HR: heart rate
HSI: heat stress index
IET: individually experienced temperature
PICO: Population/Patients, Intervention, Comparison, and Outcome
PRISMA-ScR: Preferred Reporting Items for Systematic Reviews and Meta-Analyses extension for Scoping Reviews
WBGT: wet bulb globe temperature
