# Supplementary material for: Wearables for Measuring Health Effects of Climate Change–Induced Weather Extremes: Scoping Review
Source: JMIR Mhealth Uhealth. 2022 Sep 9;10(9):e39532. doi: 10.2196/39532 (PMC9508665; doi:10.2196/39532)
Supplement: Multimedia Appendix 2 [file mhealth_v10i9e39532_app2.docx]

Table S1. Search strings (last search on 01.09.2021)

| **Database** | **Search String** |
| --- | --- |
| PubMed | ("wearabl*" OR "wearable sensor*" OR "consumer grade" OR "smartwatch*" OR "mHealth" OR "health track*" OR "fitness track*" OR "fitness watch*" OR "wristband monitor*" OR "medical internet of things" OR "activity monitor*" OR "multisensor armband" OR "wrist-worn sensor" OR "body sensor" OR "wearable electronic devices"[MeSH Terms] ) AND ("heat exposure" OR "hot temperature*"[MeSH Terms] OR "natural disasters"[MeSH Terms] OR "weather extreme" OR "extreme weather event" OR "climate change"[MeSH Terms] OR "climate change" OR "heat strain" OR "heat stress" OR "climate" OR "extreme climate" OR "hot weather" OR "global warming" OR "hot environment*" OR "warm environment" OR "heatwave" OR "heat wave" OR "flood" OR "drought*" OR "forest fire" OR "wildfire") AND ( "sleep" OR "health" OR "vital sign" OR "respiratory" OR "cardiovascular" OR "morbidity" OR "dehydration" OR "wellbeing" OR "activity" OR "productivity" OR "disease" OR "illness" OR "mental health" OR "diabetes" OR "blood sugar" OR "blood glucose" OR "asthma" OR allerg* OR "infection*" OR "infectious disease" OR "pulse" OR "heart rate" OR "respiratory rate" OR "heart beat" OR "bpm" OR "ecg" OR "electrocardiogram" OR "Atrial Fibrillation" OR "fitness" OR "walking" OR "physical activity" OR "blood pressure" OR "body temperature" OR "skin temperature" OR "blood oxygen " OR "oxygen saturation" OR "O2" OR "pulse oxymetr*" OR "capnography" OR "Heart rate variability" OR "thermal comfort" OR "heat exhaustion" OR "heat stroke" OR "heat related illness" OR "heat illness" OR "heat syncope" OR "exacerbation") |
| Web of Science | TS=(("wearabl*" OR "wearable sensor*" OR "consumer grade" OR "smartwatch*" OR "mHealth" OR "fitness track*" OR "fitness watch*" OR "wristband monitor*" OR "medical internet of things" OR "activity monitor*" OR "multisensor armband" OR "wrist-worn sensor" OR "body sensor" OR "wearable electronic device*") AND ("heat exposure" OR "hot temperature*" OR "weather extreme" OR "extreme weather event" OR "natural disaster" OR "climate change" OR "heat strain" OR "heat stress" OR "heat-related" OR "climate" OR "extreme climate" OR "hot weather" OR "global warming" OR "hot environment*" OR "warm environment" OR "heatwave" OR "heat wave" OR "flood" OR "drought*" OR "forest fire" OR "wildfire") AND ("sleep" OR "health" OR "vital sign" OR "respiratory" OR "cardiovascular" OR "morbidity" OR "dehydration" OR "wellbeing" OR "activity" OR "productivity" OR "disease" OR "illness" OR "mental health" OR "diabetes" OR "blood sugar" OR "blood glucose" OR "asthma" OR allerg* OR "infection*" OR "infectious disease" OR "pulse" OR "heart rate" OR "respiratory rate" OR "heart beat" OR "bpm" OR "ecg" OR "electrocardiogram" OR "Atrial Fibrillation" OR "fitness" OR "walking" OR "physical activity" OR "blood pressure" OR "body temperature" OR "skin temperature" OR "blood oxygen " OR "oxygen saturation" OR "O2" OR "Heart rate variability" OR "thermal comfort" OR "heat exhaustion" OR "heat stroke" OR "heat related illness" OR "heat illness" OR "heat-related injury" OR "heat syncope" OR "heat exacerbation")) |
| IEEE Xplore | ((wearabl* OR smartwatch OR mHealth OR "fitness track*" OR "fitness watch" OR "medical internet of things" OR "activity monitor" OR "body sensor") AND ("heat exposure" OR "ambient temperature" OR "hot environment" OR "hot temperature*" OR "weather extreme" OR "climate change" OR "heat strain" OR "heat stress" OR climate OR heatwave OR "high temperature") AND ( sleep OR health OR "vital sign" OR "vital parameter" OR morbidity OR wellbeing OR activity OR productivity OR disease OR illness OR "mental health" OR "body temperature" OR "thermal comfort" OR "heat exhaustion" OR "heat stroke")) |
| CINAHL | ( (wearable or smartwatch or "fitness tracker" or "body sensor") ) AND ( (heat or "climate change" or heatwave or "extreme weather" or "hot temperature" or climate or weather) ) AND ( (health or wellbeing or morbidity or illness or disease or sleep or "heat stress" or "heat illness" or "heat exhaustion" or activity or productivity or "vital signs" or "body temperature") ) |
| Scopus | TITLE-ABS-KEY(("wearabl*" OR "wearable sensor*" OR "consumer grade" OR "smartwatch*" OR "mHealth" OR "fitness track*" OR "fitness watch*" OR "wristband monitor*" OR "medical internet of things" OR "activity monitor*" OR "multisensor armband" OR "wrist-worn sensor" OR "body sensor" OR "wearable electronic device*") AND ("heat exposure" OR "hot temperature*" OR "weather extreme" OR "extreme weather event" OR "natural disaster" OR "climate change" OR "heat strain" OR "heat stress" OR "heat-related" OR "climate" OR "extreme climate" OR "hot weather" OR "global warming" OR "hot environment*" OR "warm environment" OR "heatwave" OR "heat wave" OR "flood" OR "drought*" OR "forest fire" OR "wildfire") AND ("sleep" OR "health" OR "vital sign" OR "respiratory" OR "cardiovascular" OR "morbidity" OR "dehydration" OR "wellbeing" OR "activity" OR "productivity" OR "disease" OR "illness" OR "mental health" OR "diabetes" OR "blood sugar" OR "blood glucose" OR "asthma" OR allerg* OR "infection*" OR "infectious disease" OR "pulse" OR "heart rate" OR "respiratory rate" OR "heart beat" OR "bpm" OR "ecg" OR "electrocardiogram" OR "Atrial Fibrillation" OR "fitness" OR "walking" OR "physical activity" OR "blood pressure" OR "body temperature" OR "skin temperature" OR "blood oxygen " OR "oxygen saturation" OR "O2" OR "Heart rate variability" OR "thermal comfort" OR "heat exhaustion" OR "heat stroke" OR "heat related illness" OR "heat illness" OR "heat-related injury" OR "heat syncope" OR "heat exacerbation")) |
| Ovid | 1 (wearabl* or smartwatch or fitness track* or body sensor).ti,ab,kw.  2 (heat or climate change or heatwave or extreme weather or hot  temperature).ti,ab,kw.  3 (health or wellbeing or morbidity or illness or disease or sleep or heat  stress or heat illness or heat exhaustion or activity or productivity).ti,ab,kw.  4 1 and 2 and 3  5 limit 4 to yr="2010 -Current"  Resources: Journals@Ovid Full Text September 1, 2021, Your Journals@Ovid, Books@Ovid September 1, 2021, Ovid MEDLINE(R) and Epub Ahead of Print, In-Process & Other Non-Indexed Citations, Daily and Versions(R) 1946 to September 1, 2021 |
| Google Scholar | ("climate change" OR "extreme weather" OR "heatwave" OR "heat exposure" OR "global warming" OR "hot environment") AND (wearable* OR "fitness tracker" OR smartwatch) AND (health OR sleep OR morbidity) |
