# Supplementary material for: Wearables for Measuring Health Effects of Climate Change–Induced Weather Extremes: Scoping Review
Source: JMIR Mhealth Uhealth. 2022 Sep 9;10(9):e39532. doi: 10.2196/39532 (PMC9508665; doi:10.2196/39532)
Supplement: Multimedia Appendix 3 [file mhealth_v10i9e39532_app3.docx]

Table S1. Information about the wearables.

| Study | Wearable(s) | Parameters measured with wearable(s) | | | | | | | | Wear location | | | | |
| --- | --- | --- | --- | --- | --- | --- | --- | --- | --- | --- | --- | --- | --- | --- |
|  |  | Heart rate (HR) | Physical activity | Energy expenditure | Skin temperature | Electrodermal activity | Sleep (onset, offset, duration) | Individually experienced temperature (IET) | Other* | Wristband | Chest strap | Taped to the skin | Clipped to shoe/belt/collar/bag/wristband | Other |
| Al-Bouwarthan et al. (2020) | Fitbit Flex (Fitbit, Inc.)  Polar RCX3 (Polar Electro Oy) | x | x | x |  |  |  |  |  | x | x |  |  |  |
| Al-Mohannadi et al. (2016) | Omron HJ-720 ITC pedometer (Omron Healthcare) |  | x |  |  |  |  |  |  |  |  |  |  | NA |
| AlSayed et al. (2017) | Polar H7 (connected to Polar M400) (Polar Electro Oy)  Hexoskin (Carre Technologies Inc.) | x |  |  |  |  |  |  |  | x | x |  |  | shirt |
| Bailey et al. (2019) | Thermochron iButton, Hygrochron iButton (Maxim Integrated)  HOBO Pendant (Onset Corp.) |  |  |  |  |  |  | x |  |  |  |  | x |  |
| Benita and Tuncer (2019), Benita et al. (2020) | Empatica E4 (Empatica Inc) |  |  |  | x | x |  |  |  | x |  |  |  |  |
| Benjamin et al. (2020) | Viper Pod (STATSports) | x | x |  |  |  |  |  | GPS |  | x |  |  |  |
| Bernhard et al. (2015) | HOBO Pendant (Onset Corp.) |  |  |  |  |  |  | x |  |  |  |  | x |  |
| Cedeño Laurent et al. (2018) | Basis Peak watch (Intel) | x |  |  |  |  | x |  |  | x |  |  |  |  |
| Cheong et al. (2020) | Fitbit Ionic (Fitbit, Inc.)  iButton (Maxim Integrated) | x | x |  | x |  |  | x |  | x |  |  | x |  |
| Cuddy et al. (2013) | Polar RS800CX (Polar Electro Oy) | x |  |  |  |  |  |  |  | x | x |  |  |  |
| Culp and Tonelli (2019) | Zephyr BioHarness (Medtronic) | x |  | x | x |  |  |  | RR |  | x |  |  |  |
| Edwards et al. (2015) | RT3 accelerometer (Stayhealthy) |  | x |  |  |  |  |  |  |  |  |  | x |  |
| Hamatani et al. (2017) | Microsoft Band (Microsoft) | x | x |  | x |  |  |  |  | x |  |  |  |  |
| Hass and Ellis (2019) | Hygrochron iButton (Maxim Integrated) |  |  |  |  |  |  | x |  |  |  |  | x | around the neck |
| Hondula et al. (2020) | Thermochron iButton (Maxim Integrated) |  |  |  |  |  |  | x |  |  |  |  | x |  |
| Ioannou et al. (2017) | iButton (Maxim Integrated) |  |  |  | x |  |  |  |  |  |  | x |  |  |
| Jehn et al. (2014) | accelerometer APM (Aipermon) |  | x |  |  |  |  |  |  |  |  |  | x |  |
| Kakamu et al. (2021) | LW-360HR (GISupply) | x |  | x | x |  |  |  |  | x |  |  |  |  |
| Ketko et al. (2014) | Polar heart watch (Polar RS800CX) (Polar Electro Oy) | x |  |  |  |  |  |  |  | x | x |  |  |  |
| Kim et al. (2013) | Zephyr BioHarness (Medtronic) | x |  |  |  |  |  |  | RR |  | x |  |  |  |
| Kuras et al. (2015) | Thermochron iButton (Maxim Integrated) |  |  |  |  |  |  | x |  |  |  |  | x |  |
| Lam et al. (2021) | Lifesense Mambo 2 (Lifesense)  iButton (Maxim Integrated) | x |  | x | x |  |  |  |  | x |  | x |  |  |
| Larose et al. (2014) | Polar WearLink coded (connected to Polar RS400) (Polar Electro Oy) | x |  |  |  |  |  |  |  | x | x |  |  |  |
| Lewis et al. (2016) | Actigraph GT3X+ (Actigraph LLc) |  | x |  |  |  |  |  |  |  |  |  | x |  |
| Li et al. (2020) | Polar RS800 (Polar Electro Oy) | x |  |  |  |  |  |  |  | x | x |  |  |  |
| Lisman et al. (2014) | Polar Team 2 Pro (Polar Electro Oy) | x |  |  |  |  |  |  |  |  | x |  |  |  |
| Longo et al. (2017) | Thermochron iButton (Maxim Integrated) |  |  |  |  |  |  | x |  |  |  |  | x |  |
| Lundgren et al. (2014) | Polar (model N/A) (Polar Electr Oy); LASCAR datalogger (EL-USB-2-LCD+) | x |  | x |  |  |  | x |  |  | x |  |  |  |
| MacLean et al. (2020) | iButton (Maxim Integrated)  Polar Team 2 (Polar Electro Oy) | x |  |  | x |  |  |  |  |  | x | x |  |  |
| Minor et al. (2020) | SmartBand Talk SWR30 and SWR12 (Sony) |  |  |  |  |  | x |  |  | x |  |  |  |  |
| Mitchell et al. (2018) | Actical (Philips Respironics) |  | x |  |  |  |  |  |  |  |  |  | x |  |
| Nazarian et al. (2021) | Fitbit Ionic (Fitbit, Inc.)  Hygrochron iButton, Thermochron iButton (Maxim Integrated)  Polar A300 (Polar Electro Oy) | x | x |  | x |  |  | x |  | x |  | x | x |  |
| Notley et al. (2021) | Polar M400 (Polar Electro Oy) | x |  |  |  |  |  |  |  |  | x |  |  |  |
| Ojha et al. (2020) | Empatica E4 (Empatica Inc) | x |  |  | x | x |  |  |  | x |  |  |  |  |
| Pancardo et al. (2015) | Zephyr Heart Rate Monitor (model N/A) (Medtronic) | x |  | x |  |  |  |  |  |  |  |  |  | NA |
| Quante et al. (2017) | Actigraph GT3X (Actigraph LLc) |  |  |  |  |  | x |  |  | x |  |  |  |  |
| Raval et al. (2018) | EasyLog Datalogger (EasyLog, USA) |  |  |  |  |  |  | x |  |  |  |  |  | around the neck |
| Ravanelli et al. (2015, 2016) | Polar RS 800 and Polar wearlink T31 coded (Polar Electro Oy) | x |  |  |  |  |  |  |  | x | x |  |  |  |
| Relf et al. (2018) | KuduSmart (Crossbridge Scientific Ltd.)  Polar FT1 (Polar Electro Oy) | x |  |  |  |  |  |  | LSR |  | x |  |  | upper arm |
| Relf et al. (2020) | KuduSmart (Crossbridge Scientific Ltd.)  Polar FT1 (Polar Electro Oy) | x |  |  |  |  |  |  | LSR |  | x |  |  | upper arm |
| Rosenthal et al. (2020) | Fitbit (various models) (Fitbit, Inc.) |  | x |  |  |  |  |  |  | x |  |  |  |  |
| Runkle et al. (2019), Sugg et al. (2018) | Thermochron iButton (Maxim Integrated)  Garmin Vivoactive HR (Garmin Ltd.) | x | x | x |  |  |  | x | GPS | x |  |  | x |  |
| Sahu et al. (2013) | Polar Accurex Plus (Polar Electro Oy) | x |  |  |  |  |  |  |  | x | x |  |  |  |
| Seo et al. (2016) | Zephyr BioHarness (Medtronic) | x |  |  |  |  |  |  | RR |  | x |  |  |  |
| Shakerian et al. (2021) | Empatica E4 (Empatica Inc) | x |  |  | x | x |  |  |  | x |  |  |  |  |
| Shin et al. (2015) | Actiwatch 2 (Philips Respironics); SenseWear Pro 3 (BodyMedia Inc) |  |  |  |  |  | x |  |  | x |  |  |  | upper arm |
| Suwei et al. (2019) | HOBO Pendant (Onset Corp.)  iButton (Maxim Integrated) |  |  |  |  |  |  | x |  |  |  |  | x |  |
| Uejio et al. (2018) | iButton (Maxim Integrated) |  |  |  |  |  |  | x |  |  |  |  | x |  |
| Van Hoye et al. (2014) | SenseWear Pro 3 (BodyMedia Inc)  Polar FT7 (Polar Electro Oy) | x |  | x | x | x |  | x |  |  | x |  |  | upper arm |
| Williams et al. (2019) | Basis Peak watch (Intel) | x | x |  |  | x |  |  |  | x |  |  |  |  |
| Xiong et al. (2020) | Fitbit Charge 2 (Fitbit, Inc.) |  |  |  |  |  | x |  |  | x |  |  |  |  |
| Zheng et al. (2019) | activPAL, activPAL3C (PAL Technologies) |  | x |  |  |  | x |  |  |  |  | x |  |  |
| Zhu et al. (2016) | Actiwatch 2 (Philips Respironics) |  | x |  |  |  | x |  |  | x |  |  |  |  |

*RR=respiratory rate, LSR=local sweat rate, GPS=global positioning system, NA=not applicable

Table S2. Information about the weather/climate measures.

| Study | Climate change-related weather extreme | Measured climate/weather parameter | | | | | Location where climate/weather data was obtained | | | | | |
| --- | --- | --- | --- | --- | --- | --- | --- | --- | --- | --- | --- | --- |
|  |  | Temperature | Relative humidity | Precipitation | Other* | Heat stress measure | Nearest weather station | Locally installed weather station | Sensors placed on site | Climatic chamber | Smartphone sensor | Satellite data |
| Al-Bouwarthan et al. (2020) | Heat |  |  |  |  | WBGT |  |  | x |  |  |  |
| Al-Mohannadi et al. (2016) | Heat (and precipitation) | x | x | x | x | WBGT | x |  |  |  |  |  |
| AlSayed et al. (2017) | Heat | x | x |  |  |  |  |  |  | x |  |  |
| Bailey et al. (2019) | Heat | x | x |  | x | HSI |  | x | x |  |  |  |
| Benita and Tuncer (2019), Benita et al. (2020) | Heat | x | x |  | x | WBGT,  HSI |  |  | x |  | x |  |
| Benjamin et al. (2020) | Heat | x | x |  |  | WBGT | x |  | x |  |  |  |
| Bernhard et al. (2015) | Heat | x |  |  |  |  | x |  |  |  |  |  |
| Cedeño Laurent et al. (2018) | Heat | x | x |  | x |  | x |  | x |  |  |  |
| Cheong et al. (2020) | Heat | x | x |  |  |  | x |  |  |  |  | x |
| Cuddy et al. (2013) | Heat | x | x |  |  |  |  |  |  | x |  |  |
| Culp and Tonelli (2019) | Heat |  |  |  |  | WBGT |  |  | x |  |  |  |
| Edwards et al. (2015) | Heat (and precipitation) | x |  | x | x | Heating/ cooling degrees (mean temp </> 65°F) | x |  |  |  |  |  |
| Hamatani et al. (2017) | Heat | x | x |  | x | Heat Stroke Index (using estimated core temp.) |  |  | x |  |  |  |
| Hass and Ellis (2019) | Heat | x | x |  |  | HSI | x |  |  |  |  |  |
| Hondula et al. (2020) | Heat | x |  |  |  |  | x |  |  |  |  |  |
| Ioannou et al. (2017) | Heat | x | x |  | x | UTCI | x | x |  |  |  |  |
| Jehn et al. (2014) | Heat | x | x |  | x |  | x |  |  |  |  |  |
| Kakamu et al. (2021) | Heat | x | x |  | x | WBGT,  heat stress days (Tmax≥25°C) |  |  | x |  |  |  |
| Ketko et al. (2014) | Heat | x | x |  |  |  |  |  |  | x |  |  |
| Kim et al. (2013) | Heat | x | x |  |  | HSI |  |  |  | x |  |  |
| Kuras et al. (2015) | Heat | x |  |  |  |  |  |  | x |  |  |  |
| Lam et al. (2021) | Heat | x | x |  |  | Physiological equivalent temperature (PET) |  |  | x |  |  |  |
| Larose et al. (2014) | Heat | x | x |  |  |  |  |  |  | x |  |  |
| Lewis et al. (2016) | Heat (and precipitation) | x | x | x | x | HSI | x |  |  |  |  |  |
| Li et al. (2020) | Heat | x | x |  |  |  |  |  |  | x |  |  |
| Lisman et al. (2014) | Heat | x | x |  |  |  |  |  |  | x |  |  |
| Longo et al. (2017) | Heat | x |  |  |  | Extreme heat degree minutes (EHDMs): temp. difference to 29°C | x |  |  |  |  |  |
| Lundgren et al. (2014) | Heat | x | x |  | x | Heat balance equation |  |  | x |  |  |  |
| MacLean et al. (2020) | Heat | x | x |  |  | WBGT |  |  |  | x |  |  |
| Minor et al. (2020) | Heat | x | x | x | x |  | x |  |  |  |  |  |
| Mitchell et al. (2018) | Heat |  |  |  |  | WBGT |  | x | x |  |  |  |
| Nazarian et al. (2021) | Heat | x | x |  |  | WBGT |  |  | x | x |  |  |
| Notley et al. (2021) | Heat | x | x |  |  | WBGT |  |  |  | x |  |  |
| Ojha et al. (2020) | Heat | x | x |  |  | Humidex |  |  |  | x |  |  |
| Pancardo et al. (2015) | Heat | x | x |  |  | WBGT |  |  |  |  | x |  |
| Quante et al. (2017) | Heat (and precipitation) | x |  | x | x |  | x |  |  |  |  |  |
| Raval et al. (2018) | Heat | x | x |  | x | WBGT | x |  | x |  |  |  |
| Ravanelli et al. (2015, 2016) | Heat | x | x |  |  |  |  |  |  | x |  |  |
| Relf et al. (2018) | Heat | x | x |  |  |  |  |  |  | x |  |  |
| Relf et al. (2020) | Heat | x | x |  |  |  |  |  |  | x |  |  |
| Rosenthal et al. (2020) | Wildfire |  |  |  | x |  | x |  |  |  |  |  |
| Runkle et al. (2019), Sugg et al. (2018) | Heat | x |  |  |  |  |  | x |  |  |  |  |
| Sahu et al. (2013) | Heat | x |  |  |  | WBGT |  |  | x |  |  |  |
| Seo et al. (2016) | Heat | x | x |  |  |  |  |  |  | x |  |  |
| Shakerian et al. (2021) | Heat | x | x |  |  | Humidex |  |  |  | x |  |  |
| Shin et al. (2015) | Heat | x |  |  |  |  |  |  |  | x |  |  |
| Suwei et al. (2019) | Heat | x |  |  | x | WBGT | x |  |  |  |  | x |
| Uejio et al. (2018) | Heat | x | x |  | x |  | x |  |  |  |  |  |
| Van Hoye et al. (2014) | Heat | x | x |  |  |  |  |  |  | x |  |  |
| Williams et al. (2019) | Heat | x | x |  | x |  | x |  | x |  |  |  |
| Xiong et al. (2020) | Heat | x | x |  | x |  |  |  | x |  |  |  |
| Zheng et al. (2019) | Heat (and precipitation) | x | x | x | x |  | x |  |  |  |  |  |
| Zhu et al. (2016) | Heat | x | x |  | x |  |  |  | x |  |  |  |

*Other includes: wind speed, wet bulb temperature, dry bulb temperature, dew point, mean radiant temperature, barometric pressure, visibility, CO2 concentration, air quality

Table S3. Correlation of wearables’ data and exposure to extreme weather.

| Association of heat and sleep | | |  |
| --- | --- | --- | --- |
| Study | Sleep parameter | Association | Further specification |
| Zhu et al. (2016) | Duration, efficiency, delayed onset | Negative Association | No association among participants in air-conditioned room |
| Xiong et al. (2020) | Efficiency, REM sleep percentage |  |  |
| Minor et al. (2020) | Duration, delayed onset |  | Greater for participants from lower income countries, females, older adults;  Short-term acclimatization |
| Cedeño Laurent et al. (2018) | Duration |  | Short-term acclimatization |
| Quante et al. (2019) | Duration, efficiency, midpoint time |  | In adolescents |
| Williams et al. (2019) | Disruptions | Positive association | In seniors |
| Shin et al. (2015) | Time awake during the night |  |  |
| Association of heat and physical activity | | |  |
| Study | Physical activity parameter | Association | Further specification |
| Al-Mohannadi et al. (2016) | Steps | Negative association | Greater association for females, higher age, participants from Eastern Mediterranean; negative association also for precipitation |
| Benjamin et al. (2020) | Percentage of high-speed running distance, high metabolic load distance |  | Aerobic fitness moderated the effect of WBGT on relative high-speed running distance (in female soccer players) |
| Jehn et al. (2014) | Steps |  | Worse symptoms for PAH and HI patients during heat stress |
| Edwards et al. (2015) | Moderate-vigorous physical activity, inactivity, total physical activity |  | Adjusted for age, sex, ethnicity, BMI, day length, wind speed precipitation; negative association also for precipitation |
| Lewis et al. (2016) | Moderate-vigorous physical activity, sedentary time, total physical activity |  | Maximum physical activity between 20-25°C; negative association also for precipitation |
| Zheng et al. (2019) | Moderate-vigorous physical activity |  | On weekdays; negative association also for precipitation |
| Quante et al. (2019) | Physical activity |  | Maximum physical activity at 20°C; negative association also for precipitation |
| Zheng et al. (2019) | Moderate-vigorous physical activity | Positive association | On weekends, positive association also for precipitation |
| Association of air quality during wildfires and physical activity | | | |
| Study | Physical activity parameter | Association | Further specification |
| Rosenthal et al. (2020) | Steps | Negative association |  |
| Association of heat and HR | | | |
| Study | Heart rate parameter | Association | Further specification |
| Al Sayed et al. (2017) | Mean HR | Positive association | In young, male adults |
| Larose et al. (2014) | HR |  | In middle-aged, older groups |
| Ravanelli et al. (2015, 2016) | HR |  | Electric fan use could significantly delay HR elevations |
| Cuddy et al. (2015) | HR |  | Significant difference between “at-risk” and “not at risk” group |
| Ketko et al. (2014) | HR |  | Significant higher HRs for “heat intolerant” participants |
| Lisman et al. (2014) | HR |  | BMI, percent body fat, sex and maximal oxygen uptake were associated with higher HR |
| Notley et al. (2021) | HR, heart rate reserve (HRR) |  | No significant difference between age groups, hypertension, and type 2 diabetes patients |
| Li et al. (2020) | HR | Null association |  |
| Ojha et al. (2020) | HR |  |  |
| Association of heat and other physical parameters | | | |
| Study | Physical parameter | Association | Further specification |
| Van Hoye et al. (2015) | Energy expenditure | Positive association | During high-intensity exercise |
| Ojha et al. (2020) | Electrodermal Activity,  skin temperature |  |  |
| Lam et al. (2021) | Metabolic rate (MR), HR |  | Significantly higher MRs for non-local students than local students; short-term acclimatization in second week |
| Benita et al. (2019, 2020) | Skin temperature, skin conductance response |  | Stress hotspots throughout the walking route of participants in the city |
| Cheong et al. (2020) | Skin temperature, anxiety level, HR |  | correlation between higher HR, near-body temperature, and outside temperature when participants reported mild anxiety |
| Occupational heat stress | | | |
| Study | Physical parameter | Association | Additional findings |
| Al-Bouwarthan et al. (2020) | Cardiovascular strain (HRR) | Positive association | heat stress exposure (HSE) was a stronger predictor of HRR than energy expenditure |
| Sahu et al. (2013) | Maximum HR, HR recovery |  |  |
| Culp et al. (2019) | HR, respiratory rate (RR), skin temperature |  | participants with higher BMIs had higher skin temperatures |
| Runkle et al. (2019), Sugg et al. (2018) | HR, IET |  | lower BMI, heat symptoms the week prior and the perception of heat as an occupational hazard were associated with lower heat strain risk while higher education, having a place to cool off and higher IET were associated with higher heat strain |
| Ioannou et al. (2017) | Skin temperature |  |  |
| Lundgren et al. (2014) | MR |  | Higher heat strain in females than males |
| Mitchell et al. (2018) | Physical activity | Negative association | female sex and higher age were independently associated with the minutes spent in moderate or high activity, whereas incentivized pay and multi-task work were positively associated with mean physical activity |
| Kakamu et al. (2021) | HR, EE, skin temperature | Null association |  |
